# Subcellular localization and expression of bamboo mosaic virus satellite RNA-encoded protein

**DOI:** 10.1099/vir.0.004994-0

**Published:** 2009-02

**Authors:** Paramasivan Vijaya Palani, Morgan Chiu, Wei Chen, Ching-Chi Wang, Choy-Chieng Lin, Chuen-Chi Hsu, Chi-Ping Cheng, Chung-Mong Chen, Yau-Heiu Hsu, Na-Sheng Lin

**Affiliations:** 1 Institute of Plant and Microbial Biology, Academia Sinica, Taipei, Taiwan 115, ROC; 2 Graduate Institute of Biotechnology, National Chung-Hsing University, Taichung, Taiwan 402, ROC; 3 Agricultural Biotechnology Research Center, Academia Sinica, Taipei, Taiwan 115, ROC

## Abstract

The satellite RNA of bamboo mosaic virus (satBaMV) has a single open reading frame encoding a non-structural protein, P20, which facilitates long-distance movement of satBaMV in BaMV and satBaMV co-infected plants. Immunohistochemistry and immunoelectron microscopy revealed that the P20 protein accumulated in the cytoplasm and nuclei in co-infected cells. P20 and the helper virus coat protein (CP) were highly similar in their subcellular localization, except that aggregates of BaMV virions were not labelled with anti-P20 serum. The BaMV CP protein was fairly abundant in mesophyll cells, whilst P20 was more frequently detected in mesophyll cells and vascular tissues. The expression kinetics of the P20 protein was similar to but slightly earlier than that of CP in co-infected *Bambusa oldhamii* protoplasts and *Nicotiana benthamiana* leaves. However, satBaMV-encoded protein levels declined rapidly in the late phase of co-infection. During co-infection, in addition to the intact P20, a low-molecular-mass polypeptide of 16 kDa was identified as a P20 C-terminally truncated product; the possible method of generation of the truncated protein is discussed.

## INTRODUCTION

Single-stranded satellite RNAs (satRNAs) of plant viruses, ranging from 0.2 to 1.5 kb, are classified on the basis of their coding capacity. Members of the mRNA-type satRNAs are 0.7–1.5 kb in size and contain a functional open reading frame (ORF) that encodes a non-structural protein of 20–48 kDa (Fritsch *et al.*, 1993; Lin & Hsu, 1994; Mayo *et al.*, 1999). Most of the mRNA-type satRNAs are associated with nepoviruses (Roossinck *et al.*, 1992; Fritsch *et al.*, 1993), and their non-structural proteins are indispensable for replication (Hans *et al.*, 1993; Hemmer *et al.*, 1993; Liu & Cooper, 1993). satRNAs without a functional ORF are 0.2–0.5 kb in size, with highly specialized structures, and are associated with several plant virus groups, including cucumovirus, nepovirus and sobemovirus (Roossinck *et al.*, 1992; Garcia-Arenal & Palukaitis, 1999; Simon *et al.*, 2004).

Although the ORFs of mRNA-type satRNAs are translatable, only a few reports of such translation *in vitro* and *in vivo* are available. Grapevine fanleaf virus satRNA was found to be translatable *in vitro* with wheat germ extract (WGE) and rabbit reticulocyte lysate (RRL), and in infected *Chenopodium quinoa* (Pinck *et al.*, 1988; Hans *et al.*, 1992; Moser *et al.*, 1992). Translation of the satRNA of tomato black ring virus (TBRV) with WGE and RRL resulted in the synthesis of a 48 kDa polypeptide, in both the presence and absence of helper virus RNAs (Fritsch *et al.*, 1978, 1980; Greif *et al.*, 1990; Hemmer *et al.*, 1993). A similar-sized polypeptide was detected in tobacco protoplasts infected with the TBRV isolate possessing the satRNA (Fritsch *et al.*, 1978). When arabis mosaic virus (ArMV) satRNA was translated in RRL, a single translation product of 39 kDa was detected (Liu & Cooper, 1993). Two products of 45 and 40 kDa were translated from blackcurrant reversion virus satRNA *in vitro* (Latvala-Kilby *et al.*, 2000). As yet, no report exists on the *in vivo* detection of polypeptides encoded by the satRNAs of nepoviruses ArMV and TBRV.

Bamboo mosaic virus (BaMV)-associated satRNA (satBaMV) is the only satRNA found in the potexvirus group (Lin & Hsu, 1994) and is one of the mRNA-type satRNAs. The helper virus BaMV has a single-stranded, positive-sense RNA genome of 6.4 kb with five genes (Lin *et al.*, 1992, 1994; Yang *et al.*, 1997). The 5′-proximal ORF encodes the core components of the viral replicase, the middle triple gene block encodes the movement proteins (MPs) and the 3′-proximal ORF encodes the coat protein (CP) that encapsidates the BaMV and satBaMV RNAs separately (Lin & Hsu, 1994; Lin *et al.*, 1994; Liu *et al.*, 1997). Similar to other potexviruses, BaMV has triple-gene-block proteins and a CP implicated in local and systemic movement (Chang *et al.*, 1997; Lin *et al.*, 2004, 2006).

satBaMV RNA is a single-stranded, linear molecule of 836 nt excluding the poly(A) tail and contains an ORF that encodes a non-structural polypeptide of 20 kDa, P20. The coding region is flanked by a leader of 159 nt and a 3′-terminal untranslated region of 125 nt. Unlike the nepovirus satRNA-encoded proteins (Hans *et al.*, 1993; Hemmer *et al.*, 1993; Liu & Cooper, 1993), P20 is not essential for satBaMV replication (Lin *et al.*, 1996). satBaMV depends completely on BaMV for replication and encapsidation; however, it has only low similarity with BaMV genomic RNA, except at the 5′ terminus (Lin & Hsu, 1994; Lin *et al.*, 1994). Although satBaMV resembles large nepovirus satRNAs in messenger activity, it does not share significant nucleotide or amino acid sequence similarity with any of the nepovirus satRNAs. P20 resembles the mRNA-type satRNA-encoded proteins in having a basic N-terminal domain with a strong affinity for RNA (Fritsch *et al.*, 1993; Kreiah *et al.*, 1993; Lin & Hsu, 1994; Tsai *et al.*, 1999). However, P20 shares 46 % amino acid identity with the CP of the satellite virus of panicum mosaic virus (SPMV) (Liu & Lin, 1995). SPMV CP, which is not essential for SPMV replication (Qiu & Scholthof, 2000), is implicated in encapsidation, exacerbation of symptoms in co-infected plants (Scholthof, 1999; Qiu & Scholthof, 2001), stabilization of the RNA genome, interference with a suppressor of virus-induced gene silencing (Qiu & Scholthof, 2004) and systemic invasion (Omarov *et al.*, 2005). satBaMV P20 also has a role in satBaMV systemic movement during BaMV and satBaMV co-infection (Lin *et al.*, 1996; Palani *et al.*, 2006). In addition, P20 exhibits a strong self-interaction *in vitro* and *in vivo*, and efficient cell-to-cell movement in *Nicotiana benthamiana* leaves (Palani *et al.*, 2006). The N-terminal arginine-rich motif of P20 potentiates these activities as well as intracellular targeting (Palani *et al.*, 2006). In this regard, P20 shares common biological properties with other viral MPs (Carvalho & Lazarowitz, 2004; Hsu *et al.*, 2004; Isogai & Yoshikawa, 2005). In BaMV and satBaMV co-infected *N. benthamiana* leaves, accumulation of P20 and a serologically related polypeptide of a low molecular mass of 16 kDa (P16) has been detected (Palani *et al.*, 2006). Here, we extensively analysed the subcellular localization and expression of P20 during co-infection, and identified P16 and discussed its generation.

## METHODS

### Plant and virus materials.

Leaves of BaMV-V (satBaMV-associated)-infected common bamboo (*Bambusa vulgaris* McClure) (Lin & Hsu, 1994), BaMV-O (satBaMV-free)-infected green bamboo (*Bambusa oldhamii* Munro) (Lin *et al.*, 1994) and virus-free common bamboo and green bamboo (controls) were used for microscopy.

### Immunohistochemistry and immunoelectron microscopy (IEM).

Young and rolled leaves of bamboo as described above were processed for immunohistochemistry and IEM (Lin & Langenberg, 1983; Jackson, 1992). For tissue embedding, leaf pieces (2–3×1–2 mm) were fixed in fresh 2 % formaldehyde/0.1 % glutaraldehyde, dehydrated in ethanol, infiltrated with 1 : 1 (w/v) Paraplast-Plus chips (Oxford Labware) and Histoclear (National Diagnostics) and embedded in Paraplast. Slides of paraffin sections were deparaffinized, rehydrated and exposed for 5 min to undiluted normal goat serum to block non-specific binding, before being reacted with 1 : 5000 dilutions of rabbit anti-BaMV CP (Lin & Chen, 1991) or anti-P20 (Palani *et al.*, 2006) in 0.1 M PBS for 30 min at room temperature. After three rinses in PBS, the sections were incubated in gold-labelled goat anti-rabbit IgG complexes for 1 h, rinsed again three times in PBS, washed with water and then silver-enhanced. The reaction was stopped by immersion in water.

For IEM, leaf tissues were prepared and embedded in Lowicryl HM20 as described previously (Lin & Langenberg, 1983). BaMV CP and satBaMV P20 were detected with rabbit anti-BaMV CP and anti-P20 sera, respectively, followed by gold-labelled antibody labelling. For negative controls, the same procedures were followed except that the primary antibody was replaced with pre-immune serum.

### Viral RNA and satBaMV transcripts.

BaMV RNA was prepared from BaMV-S infectious cDNA clone pCB (GenBank accession no. AF018156) as described previously (Lin *et al.*, 2004). Plasmid pBSF4 containing the full-length cDNA of prototype satBaMV (GenBank accession no. NC_003497) (Lin *et al.*, 1996) was used to prepare satBaMV transcripts. Plasmid pBSF4 was linearized with *Xba*I and transcribed with a 5′ cap structure (m7GpppG) *in vitro* using T7 RNA polymerase as described previously (Lin *et al.*, 1996). The quantity and quality of the viral RNA and synthesized transcripts were verified by agarose gel electrophoresis followed by ethidium bromide staining.

### Isolation of protoplasts, electroporation and plant inoculation.

Protoplasts were isolated from cell suspensions of *N. benthamiana* (Lin *et al.*, 1992) and *B. oldhamii* Munro (Huang *et al.*, 1990). Approximately 2×10^5^ protoplasts were electroporated with BaMV viral RNA or a mixture of viral RNA and satBaMV transcripts as described in Lin *et al.* (1996) using a Precision Pulser (BTX ECM630) equipped with an electrode at 250 V, 200 Ω and 50 μF.

One-month-old *N. benthamiana* plants were used for inoculation. Three leaves of each plant were mechanically inoculated with 0.1 μg viral RNA or a mixture of 0.1 μg each viral RNA and satBaMV transcripts. At different times after inoculation, total RNA and proteins were prepared for Northern and Western blot analyses, respectively.

### Production and specificity of monoclonal antibodies (mAbs).

Ten milligrams of peptide N20 or C21 representing aa 1–20 or 163–183 of P20 (Lin & Hsu, 1994), respectively, was mixed first with keyhole limpet haemocyanin (KLH; Sigma) in PBS at room temperature, followed by 0.6 % glutaraldehyde, and exhaustively dialysed in PBS at 4 °C. Recombinant P20 (rP20) (Tsai *et al.*, 1999) and conjugates N20–KLH and C21–KLH were used to immunize BALB/c mice [3–5 mg (kg body weight)^−1^] by intraperitoneal injection. Hybridoma lines of spleen cells of the immunized mice and NS-1 myeloma cells were generated (Galfre & Milstein, 1981) and implanted in the peritoneal cavity of a mouse. Whole IgG fractions were purified from the collected ascites fluid with an antibody purification kit (Montage; Millipore).

The immunospecificities of the mAbs against P20 (mAb-P20), N20 (mAb-N20) and C21 (mAb-C21) were verified by ELISA (Lommel *et al.*, 1982) and Western blot analysis using rP20, rP18 (*Escherichia coli*-expressed recombinant polypeptide representing the C-terminal 168 aa of P20) (Tsai *et al.*, 1999) and crude protein extracts of BaMV and satBaMV co-infected *N. benthamiana* leaves. Protein extracts from *E. coli* harbouring empty vector pET-21 and healthy *N. benthamiana* leaf extracts were used as control antigens.

### Extraction of RNA and Northern blot analysis.

Total RNA was extracted from the transfected protoplasts (Lin *et al.*, 1992) or infected leaves (Verwoerd *et al.*, 1989) for Northern blotting (Lin *et al.*, 1992). RNA from uninfected protoplasts or leaves served as negative controls. BaMV viral RNA and satBaMV positive-strand transcripts were used as positive controls.^32^P-labelled BaMV CP- and satBaMV-specific probes were prepared from pBaHB (Lin *et al.*, 1993) and pBSHE (Lin *et al.*, 1996), respectively. After probing, hybridization signals were detected using a PhosphorImager (Molecular Dynamics).

### Extraction of total protein and Western blot analysis.

Total proteins were extracted from transfected protoplasts or infected leaves (Palani *et al.*, 2006), separated by 12.5 % SDS-PAGE and electrotransferred to PVDF membranes (Immobilon-P; Millipore). The blot was treated with rabbit polyclonal anti-P20 (Palani *et al.*, 2006) or anti-BaMV CP (Lin & Chen, 1991) serum, or with mAb-P20, mAb-N20 or mAb-C21, followed by incubation with horseradish peroxidase (HRP)-conjugated anti-rabbit IgG or anti-mouse IgG. P20 or CP was detected with an ECL-Plus chemiluminescence system (Amersham) as recommended by the manufacturer. Protein concentration was determined by the Bradford method (Bradford, 1976) using Bradford dye (Bio-Rad) and BSA as a standard.

### Epitope mapping.

The epitopes of mAb-P20, mAb-N20 and mAb-C21 were mapped by detecting their individual reactions with sequence-overlapping P20 peptides (see Fig. 6) on solid-phase peptide arrays. Interactions were recognized by HRP-conjugated anti-mouse IgG and detected by the ECL-Plus system as described above.

### Analysis of the satBaMV-coding region.

The nucleotide sequence of satBaMV was compared with those of nepovirus satRNAs using the Genetics Computer Group sequence analysis program package (Wisconsin Package version 10.3; Accelrys). The bestfit program was used for amino acid pairwise comparisons. The satBaMV sequence was analysed for ORFs with the ORF Finder software package (www.ncbi.nlm.nih.gov/gorf/gorf and www.bioinformatics.org/sms/orf_find.html).

## RESULTS

### Subcellular localization of P20 and CP in BaMV and satBaMV co-infected bamboo leaves

**Fig. 1. f1:**
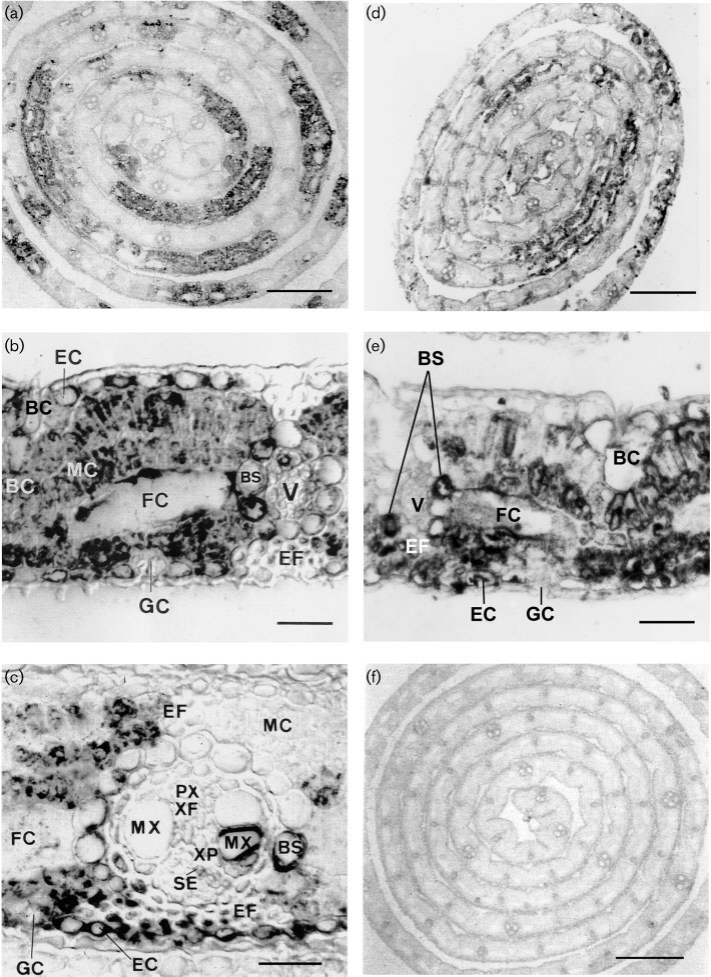
Immunogold silver staining of CP and P20 in paraffin-embedded BaMV-V-infected leaves of common bamboo (*B. vulgaris* McClure). Deparaffinized sections were treated with 1 : 5000-diluted rabbit anti-BaMV CP (a–c) (Lin & Chen, 1991), anti-P20 (d, e) (Palani *et al.*, 2006) or pre-immune (f) serum, followed by gold-labelled goat anti-rabbit IgG complexes and silver enhancement. Sections were photographed under a light microscope. Bars, 100 μm (a, d, f) or 10 μm (b, c, e). BC, Bulliform cell; BS, bundle sheath; EC, epidermal cell; EF, bundle sheath extension fibre; FC, fusoid cell; GC, guard cell; MC, mesophyll cell; MX, metaxylem; PX, protoxylem; SE, sieve elements; V, vascular bundle; XF, xylem fibre; XP, xylem parenchyma cells.

Immunodetection using rabbit anti-BaMV CP serum revealed uneven distribution of CP, with a mosaic-like pattern, in infected leaves of common bamboo (Fig. 1a[Fig f1]). CP accumulated largely in leaf mesophyll cells (Fig. 1b[Fig f1]). Fusoid cells, characterized by fusiform, colourless and thin-walled cells on both sides of the vascular bundles (Vieira *et al.*, 2002), showed a high degree of CP accumulation (Fig. 1b, c[Fig f1]). CP was also often detected in epidermal cells, bundle sheaths and bundle sheath extension fibres, and occasionally observed in metaxylem, xylem parenchyma cells, xylem fibres, sieve elements and companion cells. Bulliform cells, specialized epidermal cells, also accumulated high levels of CP; however, silver aggregates were never detected in the guard cells (Fig. 1b, c[Fig f1]). Rabbit polyclonal anti-P20 serum revealed a similar mosaic-like pattern of unevenly distributed P20 in the co-infected common bamboo leaves (Fig. 1d[Fig f1]). P20 was detected more frequently in mesophyll cells, bundle sheaths and extension fibres, and less in epidermal cells, including bulliform cells and fusoid cells. No P20 labelling was ever observed in guard cells (Fig. 1e[Fig f1]) or in BaMV-O-infected green bamboo leaves (data not shown). Pre-immune serum used as a control revealed no specific labelling (Fig. 1f[Fig f1]).

**Fig. 2. f2:**
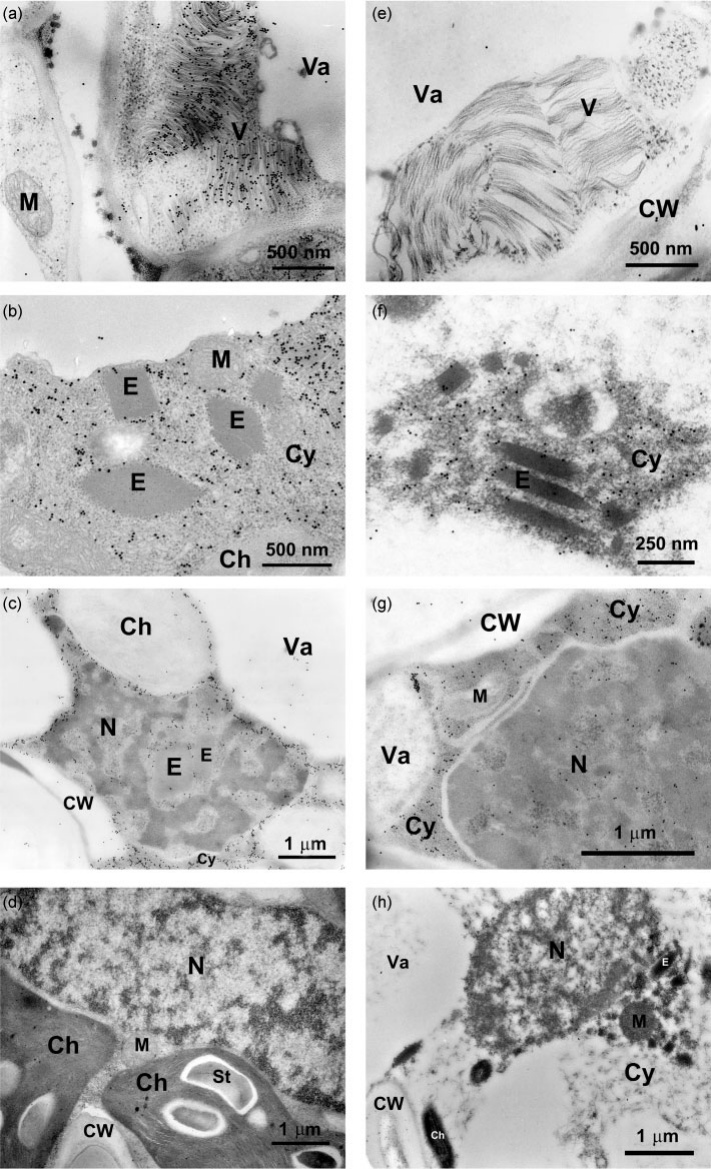
Electron micrographs of immunogold-labelled CP and P20 in glutaraldehyde-fixed and Lowicryl HM20-embedded BaMV-V-infected common bamboo (*B. vulgaris* McClure) leaves (a–c, e–g) and BaMV-O-infected green bamboo (*B. oldhamii* Munro) leaves (h). Healthy common bamboo leaves were used as a control (d). Ultrathin sections were first treated with diluted rabbit anti-BaMV CP (a–d) or anti-P20 (e–h) serum, followed by gold-labelled goat anti-rabbit IgG complexes. Ch, Chloroplast; CW, cell wall; Cy, cytoplasm; E, electron-dense crystalline bodies; M, mitochondrion; N, nucleus; St, starch; V, virion; Va, vacuole.

For IEM, immunogold labelling, with anti-BaMV CP as the primary antibody, specifically showed BaMV virions in the cytoplasm of infected leaves of common bamboo (Fig. 2a[Fig f2]). Immunogold labelling was also observed at the periphery of electron-dense crystalline bodies or inclusion bodies of TGBp1 protein (Chang *et al.*, 1997), and in both the cytoplasm and within the nucleus (Fig. 2b, c[Fig f2]). Moreover, CP was frequently detected within the infected nuclei, preferentially located in the euchromatin (Fig. 2c[Fig f2]; Lin & Chen, 1991). In contrast, BaMV virions were not labelled with anti-P20 serum (Fig. 2e[Fig f2]). P20 was detected in the cytoplasm in a free form or at the periphery of electron-dense crystalline bodies (Fig. 2f[Fig f2]) and in euchromatin of infected nuclei (Fig. 2g[Fig f2]). Anti-P20 serum revealed no specific labelling in cells infected with BaMV-O, which was free of satBaMV (Fig. 2h[Fig f2]). Anti-BaMV CP (Fig. 2d[Fig f2]) or anti-P20 sera (data not shown) labelling in healthy common bamboo or green bamboo revealed no significant labelling.

**Fig. 3. f3:**
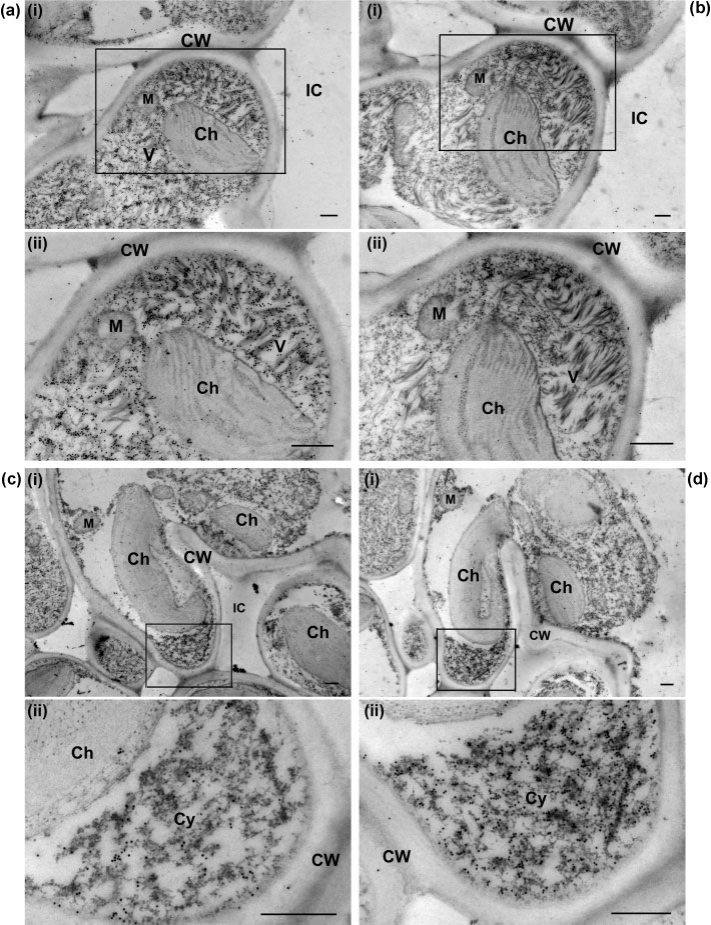
Localization of CP and P20 in serial sections of BaMV-V-infected common bamboo. Serial sections were treated with anti-BaMV-CP (a, c) or anti-P20 (b, d) serum, respectively, followed by immunogold labelling. Panels labelled (ii) show higher magnifications of the inserts in the respective panels. IC, Intercellular space; see Fig. 2 for other abbreviations.

In the serial sections of BaMV-V-infected bamboo leaves, anti-BaMV CP and anti-P20 sera revealed the different labelling abundance of corresponding proteins in the same infected cells. For example, in two adjacent sections, anti-BaMV CP labelled large aggregates of virions in the epidermal cells and mesophyll cells (Fig. 3a[Fig f3]) from which little P20 protein was detected by anti-P20 serum (Fig. 3). In contrast, anti-P20 showed strong labelling in the cytoplasm of infected phloem parenchyma and the bundle sheath extension (Fig. 3d[Fig f3]), whilst much less CP protein was detected in such cells (Fig. 3c[Fig f3]).

### Expression of P20 in BaMV and satBaMV co-infected *B. oldhamii* protoplasts

**Fig. 4. f4:**
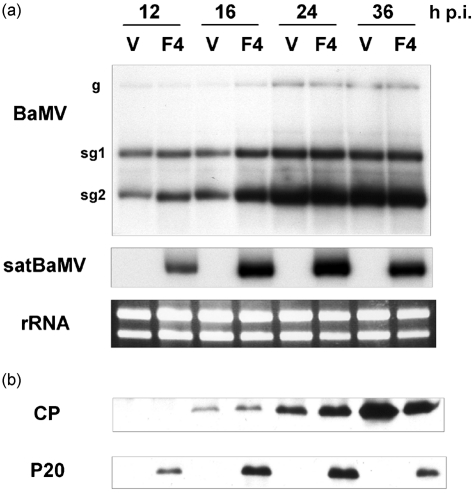
Accumulation of BaMV and satBaMV RNAs and encoded proteins in *B. oldhamii* protoplasts infected with BaMV alone (V) or co-infected with satBaMV transcripts (F4). (a) Northern blot analysis of BaMV and satBaMV RNAs. At the times indicated, total RNAs were isolated from an equal number of protoplasts, glyoxalated, electrophoresed in a 1 % agarose gel, blotted onto a nylon membrane and probed with ^32^P-labelled BaMV-specific (Lin *et al.*, 1993) or satBaMV-specific (Lin *et al.*, 1996) probes. The positions of the BaMV genomic RNA (g), subgenomic RNAs (sg1 and sg2) and satBaMV RNA are indicated on the left. rRNA, Total RNA in ethidium bromide-stained gel showing equal loading in each lane. (b) Western blot analysis of BaMV CP and satBaMV P20. From the batch of protoplasts used in (a), total proteins were extracted, resolved by 12.5 % SDS-PAGE and blotted onto PVDF membrane. Blots were probed with rabbit polyclonal anti-BaMV CP or anti-P20 serum followed by incubation with HRP-conjugated anti-rabbit IgG. CP and P20 were detected using an ECL-Plus chemiluminescence system.

To characterize further the kinetic expression of P20 protein *in vivo*, co-infection of BaMV and satBaMV in transfected bamboo protoplasts was analysed by Northern and Western blotting at different time intervals after electroporation. Accumulation of BaMV and satBaMV RNAs increased gradually with time and peaked at 36 h post-infection (Fig. 4a[Fig f4]). RNA accumulation did not increase beyond 36 h post-infection (data not shown), which was probably due to gradual loss of protoplast viability. Accumulation of BaMV genomic RNA and two subgenomic RNAs (sg1 and sg2) in the co-infected protoplasts was not significantly different from that in the protoplasts infected only with the helper RNA (Fig. 4a[Fig f4]).

Expression of CP and P20 was analysed further by Western blotting using polyclonal anti-BaMV CP or anti-P20 serum, respectively (Fig. 4b[Fig f4]). In protoplasts infected with viral RNA or co-infected with viral RNA and satBaMV, CP expression increased from 12 to 16 h post-infection onwards. Throughout the course of infection, CP was abundantly expressed and detected as a major polypeptide. In BaMV and satBaMV co-infected protoplasts, accumulation of CP was not significantly altered. Anti-P20 serum detected P20 as a major polypeptide in the BaMV and satBaMV co-infected protoplasts. The expression pattern of P20 was similar to that of CP; however, P20 started to accumulate slightly earlier than CP in the co-infected bamboo protoplasts (Fig. 4b[Fig f4]).

### Expression of P20 in BaMV and satBaMV co-infected *N. benthamiana* leaves

**Fig. 5. f5:**
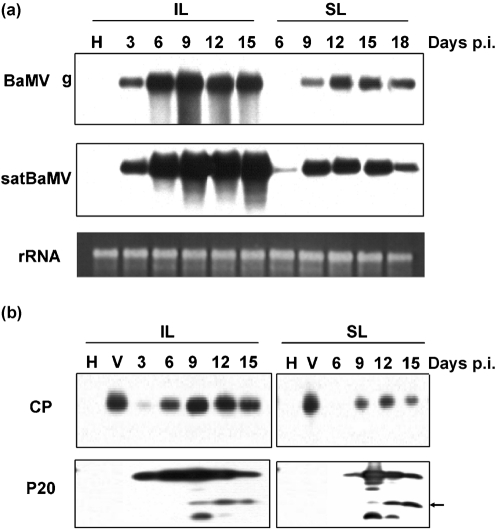
Accumulation of BaMV and satBaMV RNAs and encoded proteins in inoculated (IL) and systemic (SL) leaves of *N. benthamiana*. Northern (a) and Western (b) blot analyses of RNA and protein from leaves inoculated with BaMV (V) or co-inoculated with satBaMV transcripts at the times indicated. H, Healthy mock-inoculated leaves. Total RNA or protein was extracted from an equal amount of leaves and processed as described in the legend to Fig. 4. The position of P16 is indicated by an arrow.

In the field, bamboo plants do not usually produce seeds, and it is time-consuming to obtain virus-free bamboo seedlings through meristem-tip culture (Hsu *et al.*, 2000). Most importantly, like many woody plants, bamboo is barely susceptible to sap inoculation with BaMV. We therefore conducted co-infection studies in the experimental host *N. benthamiana*. In fact, time-course analysis of *B. oldhamii* protoplasts showed that the BaMV and satBaMV replication and protein expression kinetics were similar to those in the co-infected *N. benthamiana* protoplasts (data not shown). In BaMV and satBaMV co-infected *N. benthamiana*, P20 expression was confirmed by Northern blot analysis of BaMV and satBaMV RNAs in inoculated leaves, in which BaMV and satBaMV genomic RNAs began to accumulate at 3 days post-inoculation (p.i.). In systemic leaves, satBaMV RNA began to accumulate starting from 6 days p.i., whilst BaMV genomic RNA was detected only at 9 days p.i. (Fig. 5a[Fig f5]). Accumulation of both RNAs increased steadily with time and could be detected in both inoculated and systemic leaves up to 15–18 days p.i.

Analysis of the expression of CP and P20 proteins in inoculated leaves revealed that both proteins were detectable at 3 days p.i.; CP was only just detectable, whilst P20 was abundant (Fig. 5b[Fig f5]). Expression of both proteins peaked at 9 days p.i. and declined thereafter. In systemic leaves, P20 was detected earlier (at 6 days p.i.) than CP (9 days p.i.). As with P20 expression kinetics in inoculated leaves, the P20 level in systemic leaves peaked at 9 days p.i. and then declined. The decline phase of P20 expression began earlier than that of CP. Throughout the course of infection, in both inoculated and systemic leaves, CP was detected as a single major polypeptide. However, in both types of leaf, anti-P20 serum recognized not only a major polypeptide (P20) but also a series of polypeptides with low molecular mass. These serologically related polypeptides were predominant in the declining phase of P20 expression. At the end of P20 expression, only P20 and a polypeptide of 16 kDa (P16) were detected (Fig. 5b[Fig f5], arrow). P16 was not detected in bamboo or *N. benthamiana* protoplasts. Detection of P16 only in *N. benthamiana* leaves indicates that P16 generation is typically specific to intact plant tissue.

### Epitopes and immunospecificity of P20 mAbs

**Fig. 6. f6:**
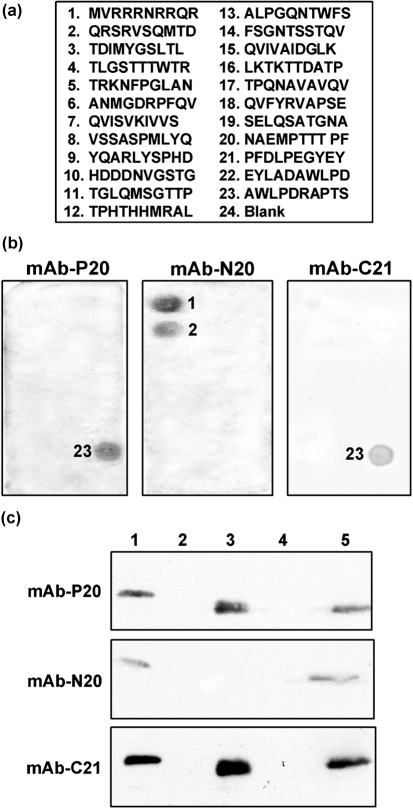
Epitope mapping and immunospecificity of P20 mAbs. (a) Overlapping amino acid sequences of satBaMV P20 used on a solid peptide array. (b) Individual interactions of mAb-P20, mAb-N20 and mAb-C21 with the P20 peptides on the solid array. HRP-conjugated anti-mouse IgG was detected by chemiluminescence. Numbers correspond to the position of the peptide on the solid array in (a). (c) Western blot analysis of the interactions of mAb-P20, mAb-N20 and mAb-C21 with rP20 (lane 1), proteins from *E. coli* harbouring empty vector pET-21 (lane 2), rP18 (lane 3), and healthy (lane 4) or satBaMV co-infected (lane 5) *N. benthamiana* leaves.

Three mAbs, mAb-P20, mAb-N20 and mAb-C21, were generated and purified to identify the P16 protein detected in the BaMV and satBaMV co-infected *N. benthamiana* leaves. The epitopes of the three mAbs were mapped using P20 peptide arrays (Fig. 6a[Fig f6]). mAb-P20 and mAb-C21 recognized a single C-terminal peptide, whereas mAb-N20 interacted with the two overlapping N-terminal P20 peptides (Fig. 6b[Fig f6]). Specific interactions of the mAbs thus indicated that mAb-P20 and mAb-C21 shared a common epitope at the P20 C terminus, encompassing aa 174–183, whereas mAb-N20 recognized aa 1–18. Similar interactions between the mAbs and peptides were also detected by ELISA (data not shown).

The immunospecificity of mAb-P20, mAb-N20 and mAb-C21 was analysed by their interaction with *E. coli*-expressed rP20, a recombinant polypeptide representing the C-terminal 168 aa of P20 (rP18) (Tsai *et al.*, 1999) and P20 expressed in co-infected *N. benthamiana* leaves (Fig. 6c[Fig f6]). All three mAbs interacted with rP20 and P20 expressed in the co-infected leaves (Fig. 6c[Fig f6], lanes 1 and 5). mAb-P20 and mAb-C21, with a common C-terminal epitope, interacted with rP18, which lacks the N-terminal 15 aa of P20 (Tsai *et al.*, 1999). mAb-N20, which interacts with an epitope at the P20 N terminus, did not interact with rP18 (Fig. 6c[Fig f6], lane 3). None of the three mAbs reacted with healthy *N. benthamiana* leaf proteins (Fig. 6c[Fig f6], lane 4).

### C-terminally truncated forms of P20 in BaMV and satBaMV co-infected *N. benthamiana* leaves

**Fig. 7. f7:**
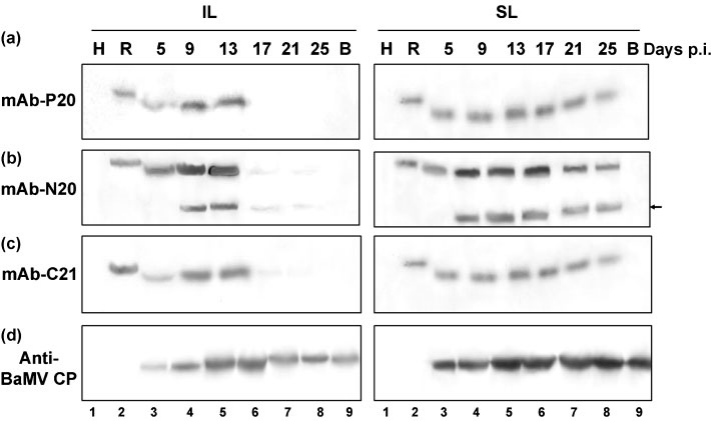
Immunodetection of satBaMV P20 and its derivatives in co-inoculated (IL) and systemic (SL) leaves of *N. benthamiana* by P20 mAbs. Total protein from healthy (lane 1), satBaMV co-infected (lanes 3–8) or only BaMV-infected (lane 9) *N. benthamiana* leaves was extracted and processed as described in Fig. 4, except that the blots were probed with the P20 mAbs mAb-P20 (a), mAb-N20 (b) and mAb-C21 (c) or with rabbit anti-BaMV CP (d). rP20 was used as a control (lane 2). The times of collection of the IL and SL leaves are indicated. The position of P16 is indicated by an arrow.

Expressed satBaMV translation products were further probed with mAb-P20, mAb-N20 and mAb-C21 to identify the nature of P20 in the BaMV and satBaMV co-infected *N. benthamiana* leaves (Fig. 7). P20 expression was abundant between 5 and 13 days p.i. with all three mAbs, but greatly declined after 17 days p.i. in the inoculated leaves (Fig. 7a–c[Fig f7]). In contrast, CP expression was consistent up to 25 days p.i. (Fig. 7d[Fig f7]). In addition to P20, a protein of molecular mass 16 kDa (P16) was detected between 9 and 13 days p.i., but only with mAb-N20 (Fig. 7b[Fig f7]). In co-infected leaves, the protein with molecular mass lower than 16 kDa that was detected by polyclonal anti-P20 serum (Fig. 5) was not detected by the mAb-N20. In upper uninoculated leaves, P20 expression was detected from 5 to 25 days p.i. with all three mAbs. Similar to its expression in inoculated leaves, P16 was recognized only by mAb-N20 from 9 to 25 days p.i. during co-infection. In both inoculated and uninoculated upper leaves, the expression pattern of P16 paralleled that of P20. Throughout the course of infection, CP was consistently detected by the antiserum against BaMV CP (Fig. 7d[Fig f7]).

## DISCUSSION

Many positive-stranded RNA viruses and viroids infect plants systemically, and their corresponding RNAs and viral CPs show uneven distribution in naturally infected plants (Lin & Langenberg, 1984; Kaufmann *et al.*, 1992; Rodio *et al.*, 2006). Our light microscopy analysis of immunostained BaMV and satBaMV co-infected bamboo leaves revealed a mosaic-like pattern of BaMV CP and satBaMV P20 expression in epidermal, mesophyll and bundle sheath cells. Both CP and P20 were predominant in the mesophyll, including in fusoid cells, the specialized cells involved in translocation and distribution of photoassimilates between the mesophyll and the vascular bundle in the Bambusoideae (Vieira *et al.*, 2002). However, no BaMV CP or P20 protein was found in the guard cells (Fig. 1) because these cells are symplasmically isolated (Ding *et al.*, 1997) and thus BaMV and satBaMV could not move from cell to cell to the guard cells.

Furthermore, at the ultrastructural level, IEM of BaMV and satBaMV co-infected bamboo leaves confirmed an almost identical subcellular localization of CP and P20, except that P20 was not detected in BaMV virions (Fig. 2e[Fig f2]). Both CP and P20 were associated with electron-dense crystalline bodies or inclusion bodies of the BaMV TGBp1 (Fig. 2b and f[Fig f2]) (Chang *et al.*, 1997); an interaction of P20 with CP and TGBp1 has been demonstrated *in vitro* and *in vivo* (Palani *et al.*, 2006; unpublished data). Furthermore, P20 with a putative nuclear localization signal was detected within the nuclei (Fig. 2g[Fig f2]). Involvement of nuclear component(s) in the transport of satBaMV ribonucleoprotein complexes needs further investigation. Although CP and TGBp1 are required for cell-to-cell and long-distance movement of BaMV and satBaMV (Lin *et al.*, 2004, 2006), P20 has been suggested to be involved in satBaMV long-distance movement (Lin *et al.*, 1996; Palani *et al.*, 2006). The arginine-rich motif of P20 is the overlapping functional domain involved in various biological activities, including RNA-binding activity (Tsai *et al.*, 1999), self-interaction, intracellular targeting and efficient cell-to-cell movement of P20 (Palani *et al.*, 2006). The viral MP properties and the concomitant accumulation of P20 in phloem parenchyma cells and bundle sheath extensions (Figs 1e[Fig f1] and 3d[Fig f3]) suggest that P20 may perform additional roles, including systemic movement of satBaMV during BaMV and satBaMV co-infection (Palani *et al.*, 2006). Likewise, SPMV CP, a P20 homologue, is known to contribute to systemic invasion of panicum mosaic virus and SPMV during co-infection (Qiu & Scholthof, 2004; Omarov *et al.*, 2005).

Besides having a similar subcellular localization, both CP and P20 exhibited similar expression kinetics in BaMV and satBaMV co-infected protoplasts and plants. The concomitant accumulation of P20 at early stages of co-infection was similar to that of other nepovirus satRNA-encoded proteins in the host (Pinck *et al.*, 1988). In addition to the intact P20, a low-molecular-mass polypeptide, P16, was detected only by mAb-N20 during the declining phase of P20 expression in *N. benthamiana*, which indicates P16 is a consequence of P20 truncation at the C terminus. IEM detection of P16 in embedded cells of the co-infected bamboo leaves was difficult, as mAb-N20 specific for a single epitope is an efficient probe only for Western blot analysis, and also because the truncated protein was detectable only in the transition phase of P20 expression in *N. benthamiana*.

As polypeptides of different sizes could be produced by RNA editing (Wu *et al.*, 1992; Maydanovych & Beal, 2006), the possibility that P16 is generated by RNA editing was analysed further in co-infected *N. benthamiana* leaves. RT-PCR revealed no truncated satBaMV RNA other than the full-length satBaMV RNA (data not shown). The absence of truncated satBaMV RNA species was also confirmed by Northern blot analysis during the time-course study (Fig. 5a[Fig f5]). satBaMV RNA editing therefore plays no role in the generation of P16, and the differential patterns of low-molecular-mass polypeptides detected in BaMV and satBaMV co-infected *N. benthamiana* leaves could be related to the time of expression.

Comparison of P20 and nepovirus satRNA-encoded proteins did not reveal any significant similarity (data not shown); however, P20 shares high sequence similarity with SPMV CP (Liu & Lin, 1995). In co-infected plants, SPMV RNA directs translation of a major polypeptide and an additional polypeptide of low molecular mass (9.4 kDa) that occurs by preferential initiation of translation from the third in-frame AUG codon (Omarov *et al.*, 2005). The functionality of the in-frame codons and additional translational products has also been reported in nepovirus satRNAs (Liu *et al.*, 1990; Latvala-Kilby *et al.*, 2000). satBaMV RNA in the first reading frame revealed the longest ORF for a polypeptide of molecular mass 20 153 Da, which was detected in the co-infected protoplasts and plants (Figs 4b[Fig f4] and 5b[Fig f5]). On the basis of prediction, there are seven in-frame AUG codons in the first reading frame of satBaMV RNA and a putative ORF in the second reading frame. Although the base sequence surrounding the third (CATCAUGT) and the fourth (CAATAUGG) AUG codons most closely fits the consensus sequence for translation proposed by Kozak (1984) and Lutcke *et al.* (1987), preferential translation may be initiated at the fourth AUG codon, which has an A at position −3 and a G at +4 (Kozak, 1984; Lutcke *et al.*, 1987), with a predicted polypeptide of 15.2 kDa. Although the molecular mass of P16 is very close to 15.2 kDa, P16 was detected in the co-infected *N. benthamiana* leaves only with the wild-type satBaMV and not with the arginine-rich motif deletion mutant of satBaMV (Palani *et al.*, 2006). Furthermore, in the RRL and WGE systems, only P20 was translated from satBaMV RNA and, in addition, a low-molecular-mass polypeptide of 18 kDa was detected (Lin & Hsu, 1994; N. S. Lin & Y. H. Hsu, unpublished data). Detection of P16 in BaMV and satBaMV co-infected leaves only by mAb-N20 (Fig. 7b[Fig f7]) suggested that the initiation of a second translation product of satBaMV RNA did not occur and that P16 is simply the C-terminally truncated version of P20. As proteolytic processing of viral proteins has implications for host–virus interactions and has a differential effect on the functionality of the proteins (Dunigan *et al.*, 1988; Hughes *et al.*, 1995; Héricourt *et al.*, 2000; Mulder & Muesing, 2000; Reichel & Beachy, 2000; Karsies *et al.*, 2001; Vierstra, 2003), the possibility of P16 generation as a consequence of P20 proteolysis during co-infection *in planta* can likewise not be eliminated.

Although we have not extensively analysed the biological significance of the P20 C terminus in BaMV and satBaMV co-infection, our replication studies of the aa 166–183 truncation mutant indicated a significant reduction in satBaMV accumulation to about 70–80 % in *N. benthamiana* leaves compared with that of the wild-type satBaMV (data not shown). Whether P16 accumulation during satBaMV co-infection reflects a strategy by which BaMV and satBaMV regulate the intracellular levels of mature P20, or is a host defence mechanism aimed at the elimination of P20, are subjects for further investigation.

## References

[r1] Bradford, M. M. (1976). A rapid and sensitive method for quantitation of microgram quantities of protein utilizing the principle of protein-dye binding. Anal Biochem 72, 248–254. 942051 10.1016/0003-2697(76)90527-3

[r2] Carvalho, M. F. & Lazarowitz, S. G. (2004). Interaction of the movement protein NSP and the *Arabidopsis* acetyltransferase AtNSI is necessary for cabbage leaf curl geminivirus infection and pathogenicity. J Virol 78, 11161–11171. 15452236 10.1128/JVI.78.20.11161-11171.2004 PMC521842

[r3] Chang, B. Y., Lin, N. S., Loiu, D. Y., Chen, J. P., Liou, G. G. & Hsu, Y. H. (1997). Subcellular localization of the 28 kDa protein of the triple-gene-block of bamboo mosaic potexvirus. J Gen Virol 78, 1175–1179. 9152438 10.1099/0022-1317-78-5-1175

[r4] Ding, B., Kwon, M. O., Hammond, R. & Owens, R. (1997). Cell-to-cell movement of potato spindle tuber viroid. Plant J 12, 931–936. 9375403 10.1046/j.1365-313x.1997.12040931.x

[r5] Dunigan, D. D., Dietzgen, R. G., Schoelz, J. E. & Zaitlin, M. (1988). Tobacco mosaic virus particles contain ubiquitinated coat protein subunits. Virology 165, 310–312. 2838968 10.1016/0042-6822(88)90691-5

[r6] Rodio, M. E., Delgado, S., Flores, R. & Serio, D. F. (2006). Variants of *Peach latent mosaic viroid* inducing peach calico: uneven distribution in infected plants and requirements of the insertion containing the pathogenicity determinant. J Gen Virol 87, 231–240. 16361436 10.1099/vir.0.81356-0

[r7] Fritsch, C., Mayo, M. A. & Murant, A. F. (1978). Translation of the satellite RNA of tomato black ring virus *in vitro* and in tobacco protoplasts. J Gen Virol 40, 587–593.

[r8] Fritsch, C., Mayo, M. A. & Murant, A. F. (1980). Translation products of genome and satellite RNAs of tomato black ring virus. J Gen Virol 46, 381–389.

[r9] Fritsch, C., Mayo, M. & Hemmer, O. (1993). Properties of the satellite RNA of nepoviruses. Biochimie 75, 561 7505622 10.1016/0300-9084(93)90062-w

[r10] Galfre, G. & Milstein, C. (1981). Preparation of monoclonal antibodies: strategies and procedures. Methods Enzymol 73, 43–46. 10.1016/0076-6879(81)73054-4 7300683

[r11] Garcia-Arenal, F. & Palukaitis, P. (1999). Structure and functional relationships of satellite RNAs of cucumber mosaic virus. Curr Top Microbiol Immunol 239, 37–63. 9893368 10.1007/978-3-662-09796-0_3

[r12] Greif, C., Hemmer, O., Demangeat, G. & Fritsch, C. (1990). *In vitro* synthesis of biologically active transcripts of tomato black ring virus satellite RNA. J Gen Virol 71, 907–915. 1691271 10.1099/0022-1317-71-4-907

[r13] Hans, F., Fuchs, M. & Pinck, L. (1992). Replication of grapevine fanleaf virus satellite RNA transcripts in *Chenopodium quinoa* protoplasts. J Gen Virol 73, 2517–2523. 1383395 10.1099/0022-1317-73-10-2517

[r14] Hans, F., Pinck, M. & Pinck, L. (1993). Location of the replication determinants of the satellite RNA associated with grapevine fanleaf nepovirus (strain F13). Biochimie 75, 597–603. 7505623 10.1016/0300-9084(93)90066-2

[r15] Hemmer, O., Oncino, C. & Fritsch, C. (1993). Efficient replication of the *in vitro* transcripts from cloned cDNA of tomato black ring virus satellite RNA requires the 48K RNA-encoded protein. Virology 194, 800–806. 7684878 10.1006/viro.1993.1321

[r16] Héricourt, F., Blanc, S., Redeker, V. & Jupin, I. (2000). Evidence for phosphorylation and ubiquitinylation of the turnip yellow mosaic virus RNA-dependent RNA polymerase domain expressed in a baculovirus-insect cell system. Biochem J 349, 417–425. 10880340 10.1042/0264-6021:3490417 PMC1221164

[r17] Hsu, Y. H., Annamalai, P., Lin, C. S., Chen, Y. Y., Chang, W. C. & Lin, N. S. (2000). A sensitive method for detecting bamboo mosaic virus (BaMV) and establishment of BaMV-free meristem tip cultures. Plant Pathol 49, 101–107.

[r18] Hsu, H. T., Hsu, Y. H., Bil, I. P., Lin, N. S. & Chang, B. Y. (2004). Biological functions of the cytoplasmic TGBp1 inclusions of bamboo mosaic potexvirus. Arch Virol 149, 1027–1035. 15098116 10.1007/s00705-003-0254-y

[r19] Huang, L. C., Huang, B.-L. & Chen, W. L. (1990). Tissue culture investigations of bamboo. V. Recovery of callus from protoplasts of suspension-cultured *Bambusa* cells. Bot Bull Acad Sin 31, 29–34.

[r20] Hughes, R. K., Perbal, M. C., Maule, A. J. & Hull, R. (1995). Evidence for proteolytic processing of tobacco mosaic virus movement protein in *Arabidopsis thaliana*. Mol Plant Microbe Interact 8, 658–665. 7579611 10.1094/mpmi-8-0658

[r21] Isogai, M. & Yoshikawa, N. (2005). Mapping the RNA-binding domain on the *Apple chlorotic leaf spot virus* movement protein. J Gen Virol 86, 225–229. 15604450 10.1099/vir.0.80493-0

[r22] Jackson, D. (1992). *In situ* hybridization in plants. In *Molecular Plant Pathology: a Practical Approach*, pp. 163–174. Edited by S. J. Gurr, M. McPherson & D. J. Bowles. Oxford: Oxford University Press.

[r23] Karsies, A., Horn, T. & Leclerc, D. (2001). Degradation signals within both terminal domains of the cauliflower mosaic virus capsid protein precursor. Plant J 27, 335–343. 11532179 10.1046/j.1365-313x.2001.01093.x

[r24] Kaufmann, A., Koenig, R. & Lesemann, D. E. (1992). Tissue print-immunoblotting reveals an uneven distribution of beet necrotic yellow vein and beet soil-borne viruses in sugarbeets. Arch Virol 126, 329–335. 1524497 10.1007/BF01309706

[r25] Kozak, M. (1984). Compilation and analysis of sequences upstream from the translational start site in eukaryotic mRNAs. Nucleic Acids Res 12, 857–872. 6694911 10.1093/nar/12.2.857 PMC318541

[r26] Kreiah, S., Cooper, J. I. & Strunk, G. (1993). The nucleotide sequence of a satellite RNA associated with strawberry latent ringspot virus. J Gen Virol 74, 1163–1165. 7685374 10.1099/0022-1317-74-6-1163

[r27] Latvala-Kilby, S., Lemmetty, A. & Lehto, K. (2000). Molecular characterization of a satellite RNA associated with blackcurrant reversion nepovirus. Arch Virol 145, 51–61. 10664405 10.1007/s007050050004

[r28] Lin, N. S. & Chen, C. C. (1991). Association of bamboo mosaic virus (BaMV) and BaMV-specific electron-dense crystalline bodies with chloroplasts. Phytopathology 81, 1551–1555.

[r29] Lin, N. S. & Hsu, Y. H. (1994). A satellite RNA associated with bamboo mosaic potexvirus. Virology 202, 707–714. 7518162 10.1006/viro.1994.1392

[r30] Lin, N. S. & Langenberg, W. G. (1983). Immunohistochemical localization of barley stripe mosaic virions in infected wheat cells. J Ultrastruct Res 84, 16–23. 6887322 10.1016/s0022-5320(83)90082-5

[r31] Lin, N. S. & Langenberg, W. G. (1984). Distribution of barley stripe mosaic virus protein in infected wheat root and shoot tips. J Gen Virol 65, 2217–2224.

[r32] Lin, N. S., Huang, T. Z. & Hsu, Y. H. (1992). Infection of barley protoplasts with bamboo mosaic virus RNA. Bot Bull Acad Sin 33, 271–275.

[r33] Lin, N. S., Chai, Y. J., Huang, T. Y., Chang, T. Y. & Hsu, Y. H. (1993). Incidence of bamboo mosaic potexvirus in Taiwan. Plant Dis 77, 448–450.

[r34] Lin, N. S., Lin, B. Y., Lo, N. W., Hu, C. C., Chow, T. Y. & Hsu, Y. H. (1994). Nucleotide sequence of the genomic RNA of bamboo mosaic potexvirus. J Gen Virol 75, 2513–2518. 8077956 10.1099/0022-1317-75-9-2513

[r35] Lin, N. S., Lee, Y. S., Lin, B. Y., Lee, C. W. & Hsu, Y. H. (1996). The open reading frame of bamboo mosaic potexvirus satellite RNA is not essential for its replication and can be replaced with a bacterial gene. Proc Natl Acad Sci U S A 93, 3138–3142. 8610182 10.1073/pnas.93.7.3138 PMC39775

[r36] Lin, M. K., Chang, B. Y., Liao, J. T., Lin, N. S. & Hsu, Y. H. (2004). Arg-16 and Arg-21 in the N-terminal region of the triple-gene-block protein 1 of *Bamboo mosaic virus* are essential for virus movement. J Gen Virol 85, 251–259. 14718640 10.1099/vir.0.19442-0

[r37] Lin, M. K., Hu, C. C., Lin, N. S., Chang, B. Y. & Hsu, Y. H. (2006). Movement of potexviruses requires species-specific interactions among the cognate triple gene block proteins, as revealed by a *trans*-complementation assay based on the bamboo mosaic virus satellite RNA-mediated expression system. J Gen Virol 87, 1357–1367. 16603539 10.1099/vir.0.81625-0

[r38] Liu, Y. Y. & Cooper, J. I. (1993). The multiplication in plants of arabis mosaic virus satellite RNA requires the encoded protein. J Gen Virol 74, 1471–1474. 7687652 10.1099/0022-1317-74-7-1471

[r39] Liu, J. S. & Lin, N. S. (1995). Satellite RNA associated with bamboo mosaic potexvirus shares similarity with satellites associated with sobemoviruses. Arch Virol 140, 1511–1514. 7544972 10.1007/BF01322678

[r40] Liu, Y. Y., Hellen, C. U., Cooper, J. I., Bertioli, D. J., Coates, D. & Bauer, G. (1990). The nucleotide sequence of a satellite RNA associated with arabis mosaic nepovirus. J Gen Virol 71, 1259–1263. 1693660 10.1099/0022-1317-71-6-1259

[r41] Liu, J. S., Hsu, Y. H., Huang, T. Y. & Lin, N. S. (1997). Molecular evolution and phylogeny of satellite RNA associated with bamboo mosaic potexvirus. J Mol Evol 44, 207–213. 9069181 10.1007/pl00006137

[r42] Lommel, S. A., McCain, A. H. & Morris, T. J. (1982). Evaluation of indirect enzyme-linked immunosorbent assay for the detection of plant viruses. Phytopathology 72, 1018–1022.

[r43] Lutcke, H. A., Chow, K. C., Mickel, F. S., Moss, K. A., Keren, H. F. & Scheele, G. A. (1987). Selection of AUG initiation codons differs in plants and animals. EMBO J 6, 43–48. 3556162 10.1002/j.1460-2075.1987.tb04716.x PMC553354

[r44] Maydanovych, O. & Beal, P. A. (2006). Breaking the central dogma by RNA editing. Chem Rev 106, 3397–3411. 16895334 10.1021/cr050314a

[r45] Mayo, M. A., Taliansky, M. E. & Jackson, A. O. (1999). Large satellite RNA: molecular parasitism or molecular symbiosis. Curr Top Microbiol Immunol 239, 65–80. 9893369 10.1007/978-3-662-09796-0_4

[r46] Moser, O., Fuchs, M., Pinck, L. & Garaud, C. S. (1992). Immunodetection of grapevine fanleaf virus satellite RNA-encoded protein in infected *Chenopodium quinoa*. J Gen Virol 73, 3033–3038. 1279107 10.1099/0022-1317-73-11-3033

[r47] Mulder, L. C. & Muesing, M. A. (2000). Degradation of HIV-1 integrase by the N-end rule pathway. J Biol Chem 275, 29749–29753. 10893419 10.1074/jbc.M004670200

[r48] Omarov, R. T., Qi, D. & Scholthof, K. B. G. (2005). The capsid protein of satellite *Panicum mosaic virus* contributes to systemic invasion and interacts with its helper virus. J Virol 79, 9756–9764. 16014937 10.1128/JVI.79.15.9756-9764.2005 PMC1181559

[r49] Palani, P. V., Kasiviswanathan, V., Chen, J. C.-F., Chen, W., Hsu, Y. H. & Lin, N. S. (2006). The arginine-rich motif of *Bamboo mosaic virus* satellite RNA-encoded P20 mediates self-interaction, intracellular targeting, and cell-to-cell movement. Mol Plant Microbe Interact 19, 758–767. 16838788 10.1094/MPMI-19-0758

[r50] Pinck, L., Fuchs, M., Pinck, M., Ravelonandro, M. & Walter, B. (1988). A satellite RNA in grapevine fanleaf virus strain F13. J Gen Virol 69, 233–239. 10.1099/0022-1317-70-4-955 2471799

[r51] Qiu, W. & Scholthof, K.-B. G. (2000). *In vitro* and *in vivo* generated defective RNAs of satellite panicum mosaic virus define *cis*-acting RNA elements required for replication and movement. J Virol 74, 2247–2254. 10666255 10.1128/jvi.74.5.2247-2254.2000 PMC111706

[r52] Qiu, W. & Scholthof, K.-B. G. (2001). Genetic identification of multiple biological roles associated with the capsid protein of satellite panicum mosaic virus. Mol Plant Microbe Interact 14, 21–30. 11194868 10.1094/MPMI.2001.14.1.21

[r53] Qiu, W. & Scholthof, K.-B. G. (2004). Satellite panicum mosaic virus capsid elicits symptoms on a nonhost plant and interferes with a suppressor of virus-induced gene silencing. Mol Plant Microbe Interact 17, 263–271. 15000393 10.1094/MPMI.2004.17.3.263

[r54] Reichel, C. & Beachy, R. N. (2000). Degradation of tobacco mosaic virus movement protein by the 26S proteasome. J Virol 74, 3330–3337. 10708450 10.1128/jvi.74.7.3330-3337.2000 PMC111834

[r55] Roossinck, M. J., Sleat, D. & Palukaitis, P. (1992). Satellite RNAs of plant viruses: structures and biological effects. Microbiol Rev 56, 265–279. 1620065 10.1128/mr.56.2.265-279.1992 PMC372867

[r56] Scholthof, K.-B. G. (1999). A synergism induced by satellite panicum mosaic virus. Mol Plant Microbe Interact 12, 163–166.

[r57] Simon, A. E., Roossinck, M. J. & Havelda, Z. (2004). Plant virus satellite and defective interfering RNAs: new paradigms for a new century. Annu Rev Phytopathol 42, 415–437. 15283672 10.1146/annurev.phyto.42.040803.140402

[r58] Tsai, M. S., Hsu, Y. H. & Lin, N. S. (1999). Bamboo mosaic potexvirus satellite RNA (satBaMV RNA)-encoded P20 protein preferentially binds to satBaMV RNA. J Virol 73, 3032–3039. 10074153 10.1128/jvi.73.4.3032-3039.1999 PMC104063

[r59] Verwoerd, T. C., Dekker, B. M. & Hoekema, A. (1989). A small scale procedure for the rapid isolation of plant RNAs. Nucleic Acids Res 17, 2362 2468132 10.1093/nar/17.6.2362 PMC317610

[r60] Vieira, R. C., Gomes, D. M. S., Sarahyba, L. S. & Arruda, R. C. O. (2002). Leaf anatomy of three herbaceous bamboo species. Braz J Biol 62, 907–922. 12659043 10.1590/s1519-69842002000500021

[r61] Vierstra, R. D. (2003). The ubiquitin/26S proteosome pathway, the complex last chapter in the life of many proteins. Trends Plant Sci 8, 135–142. 12663224 10.1016/S1360-1385(03)00014-1

[r62] Wu, H. N., Wang, Y. J., Hung, C. F., Lee, H. J. & Lai, M. M. (1992). Sequence and structure of the catalytic RNA of hepatitis delta virus genomic RNA. J Mol Biol 223, 233–245. 1731072 10.1016/0022-2836(92)90728-3

[r63] Yang, C. C., Liu, J. S., Lin, C. P. & Lin, N. S. (1997). Nucleotide sequence and phylogenetic analysis of a bamboo mosaic potexvirus isolate from common bamboo (*Bambusa vulgaris* McClure). Bot Bull Acad Sin 38, 77–84.

